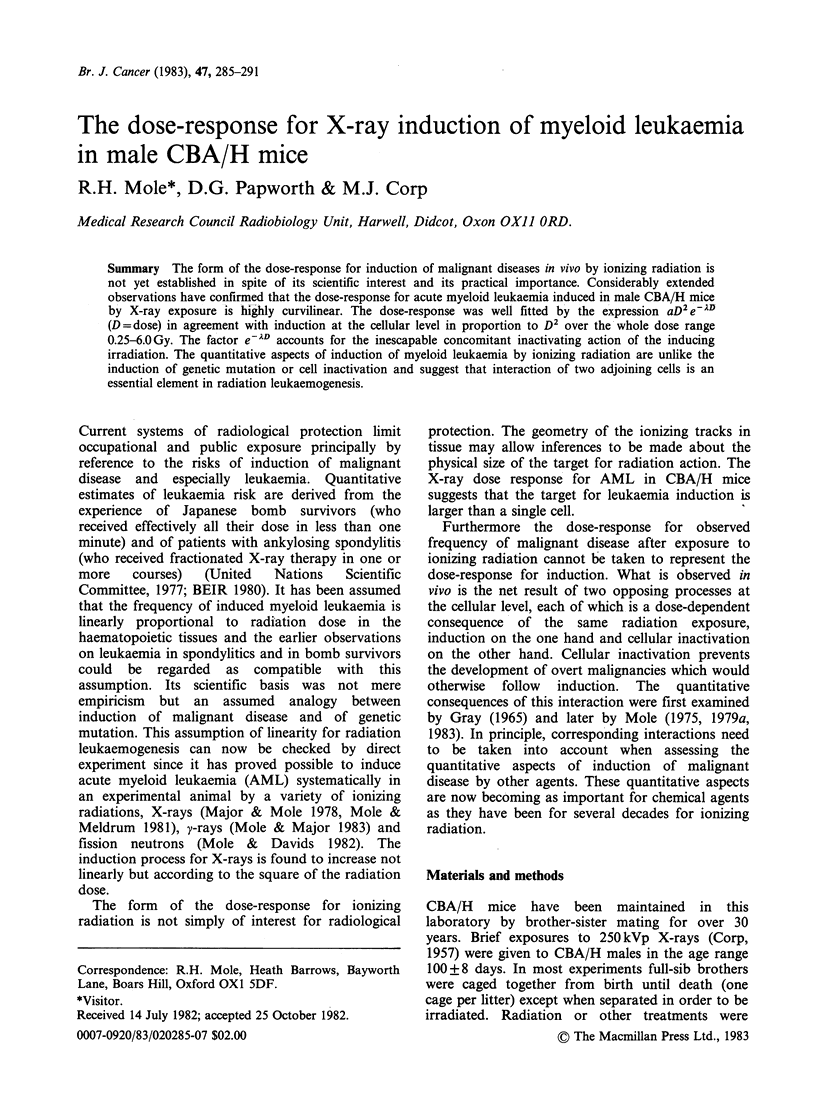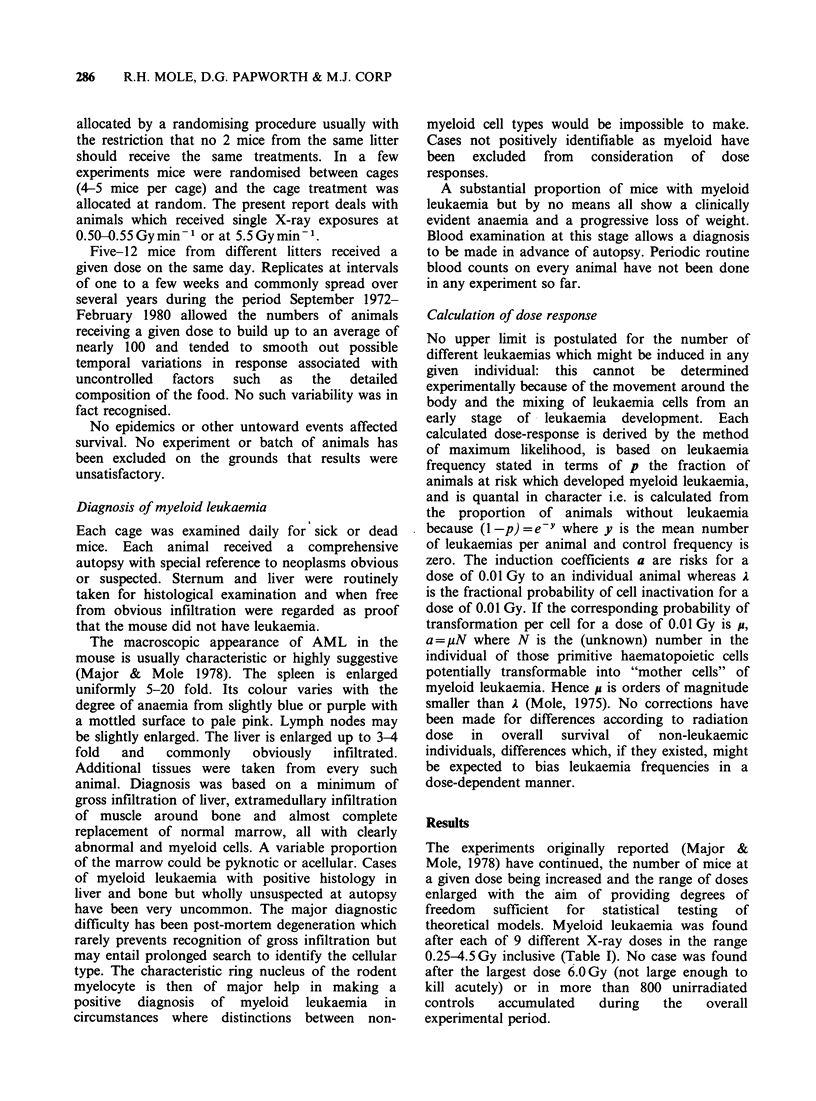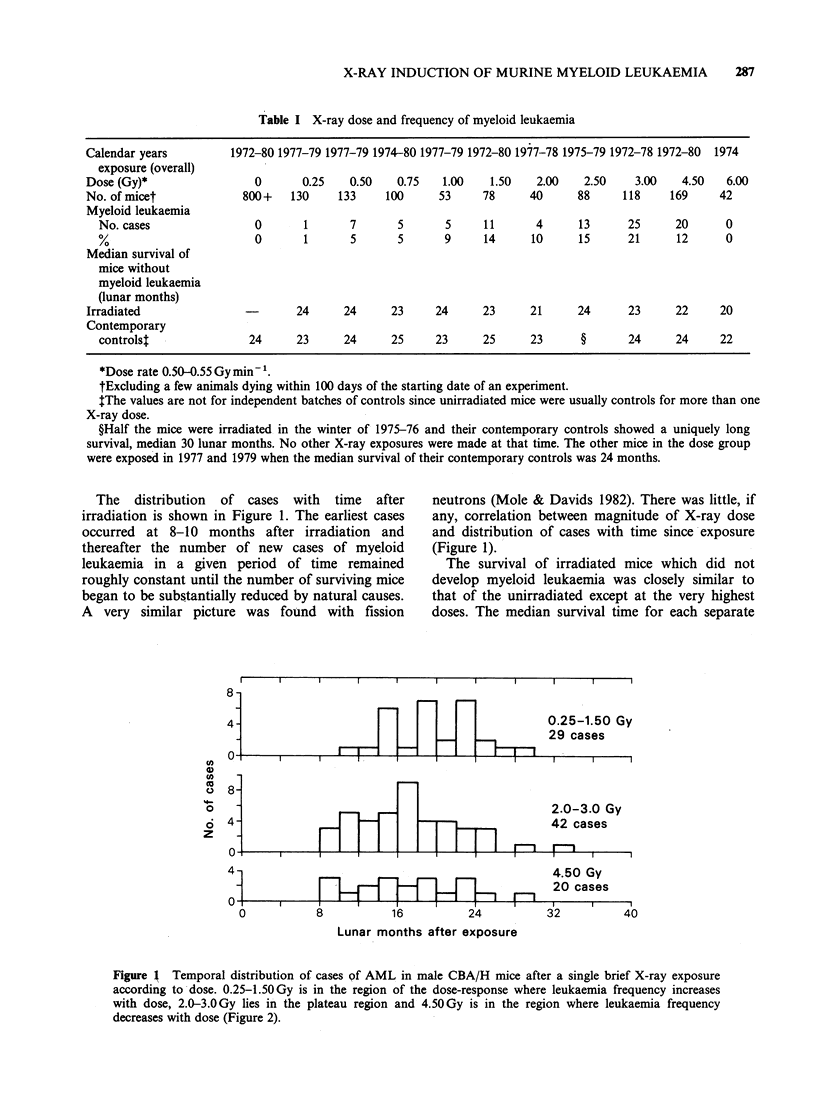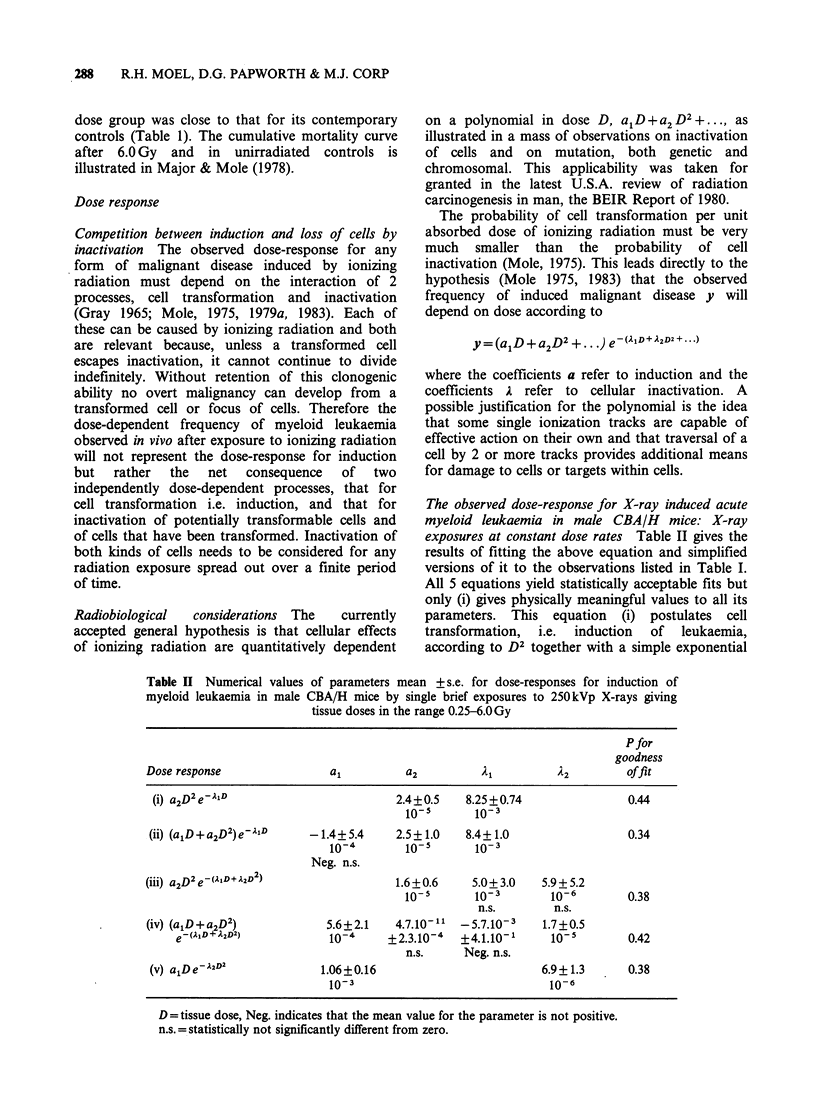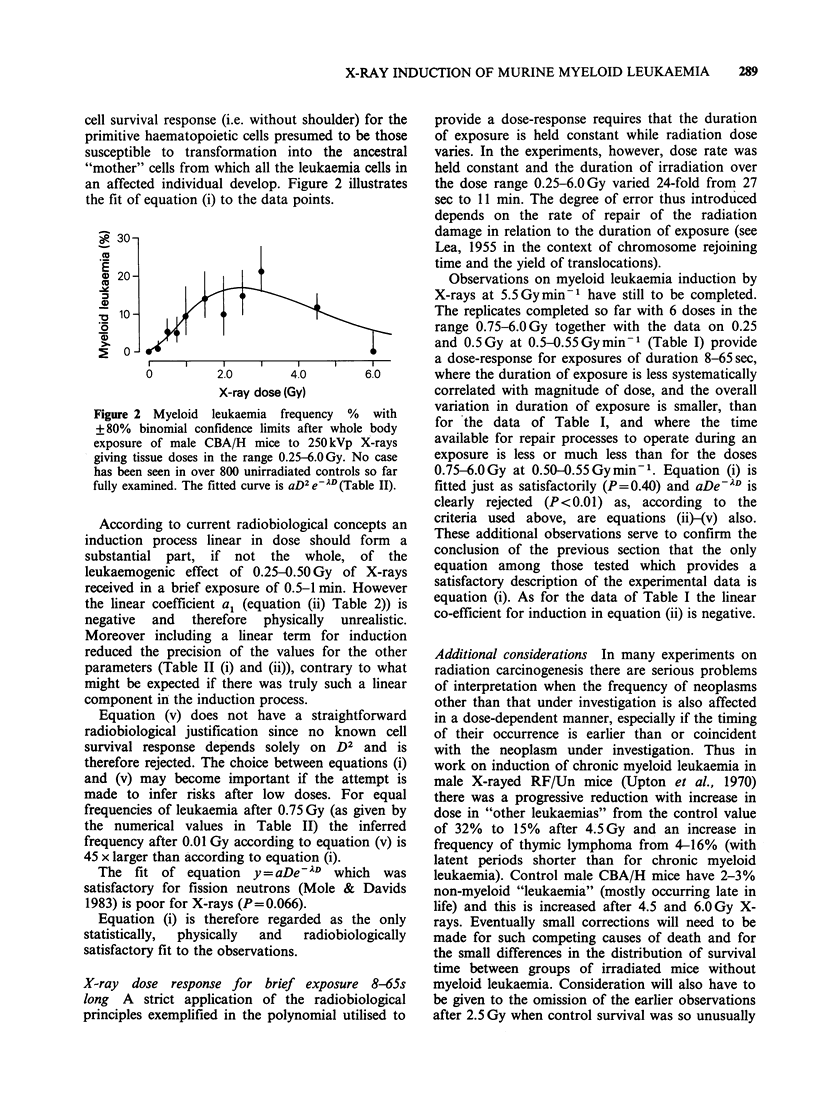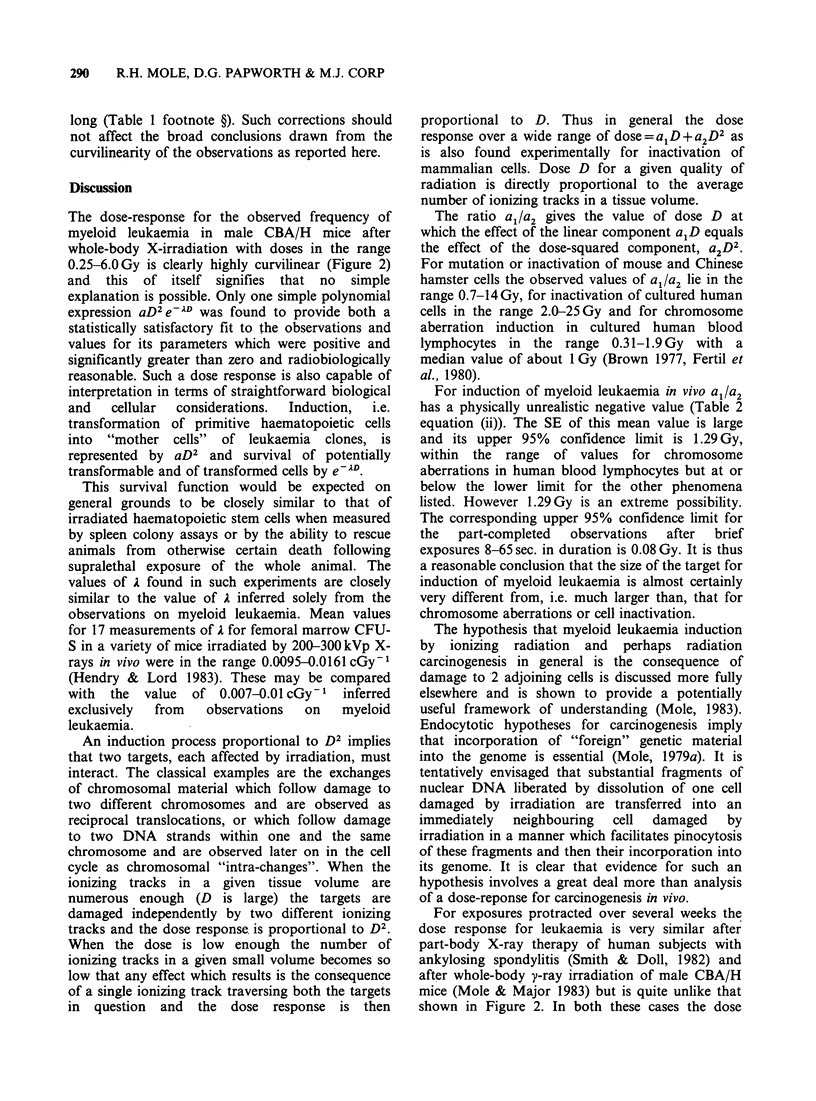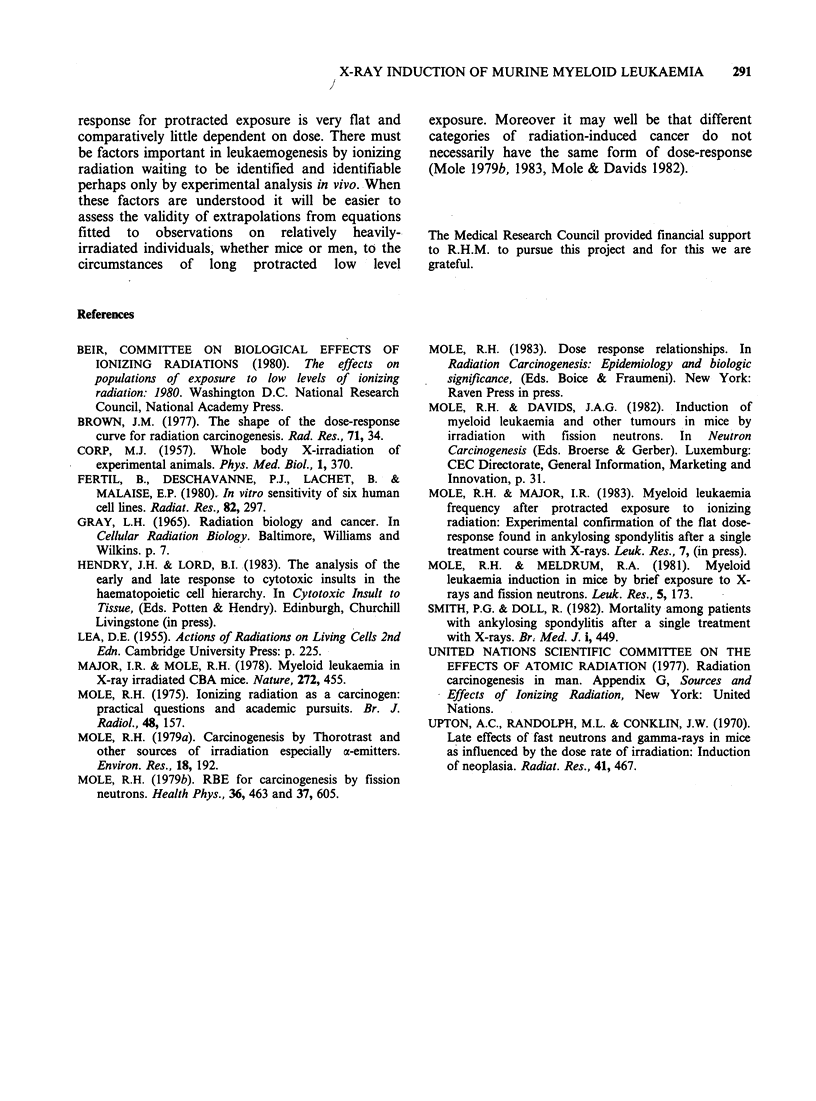# The dose-response for x-ray induction of myeloid leukaemia in male CBA/H mice.

**DOI:** 10.1038/bjc.1983.37

**Published:** 1983-02

**Authors:** R. H. Mole, D. G. Papworth, M. J. Corp

## Abstract

The form of the dose-response for induction of malignant diseases in vivo by ionizing radiation is not yet established in spite of its scientific interest and its practical importance. Considerably extended observations have confirmed that the dose-response for acute myeloid leukaemia induced in male CBA/H mice by X-ray exposure is highly curvilinear. The dose-response was well fitted by the expression aD2e-lambda D (D = dose) in agreement with induction at the cellular level in proportion to D2 over the whole dose range 0.25-6.0 Gy. The factor e-lambda D accounts for the inescapable concomitant inactivating action of the inducing irradiation. The quantitative aspects of induction of myeloid leukaemia by ionizing radiation are unlike the induction of genetic mutation or cell inactivation and suggest that interaction of two adjoining cells is an essential element in radiation leukaemogenesis.


*Br. J. Cancer* (1983), **47**, 285–291

### The dose-response for X-ray induction of myeloid leukaemia in male CBA/H mice

R.H. Mole*, D.G. Papworth & M.J. Corp

*Medical Research Council Radiobiology Unit, Harwell, Didcot, Oxon OX11 0RD.*

**Summary** The form of the dose-response for induction of malignant diseases *in vivo* by ionizing radiation is not yet established in spite of its scientific interest and its practical importance. Considerably extended observations have confirmed that the dose-response for acute myeloid leukaemia induced in male CBA/H mice by X-ray exposure is highly curvilinear. The dose-response was well fitted by the expression $aD^2e^{-\lambda D}$ ($D$=dose) in agreement with induction at the cellular level in proportion to $D^2$ over the whole dose range 0.25–6.0 Gy. The factor $e^{-\lambda D}$ accounts for the inescapable concomitant inactivating action of the inducing irradiation. The quantitative aspects of induction of myeloid leukaemia by ionizing radiation are unlike the induction of genetic mutation or cell inactivation and suggest that interaction of two adjoining cells is an essential element in radiation leukaemogenesis.

Current systems of radiological protection limit occupational and public exposure principally by reference to the risks of induction of malignant disease and especially leukaemia. Quantitative estimates of leukaemia risk are derived from the experience of Japanese bomb survivors (who received effectively all their dose in less than one minute) and of patients with ankylosing spondylitis (who received fractionated X-ray therapy in one or more courses) (United Nations Scientific Committee, 1977; BEIR 1980). It has been assumed that the frequency of induced myeloid leukaemia is linearly proportional to radiation dose in the haematopoietic tissues and the earlier observations on leukaemia in spondylitics and in bomb survivors could be regarded as compatible with this assumption. Its scientific basis was not mere empiricism but an assumed analogy between induction of malignant disease and of genetic mutation. This assumption of linearity for radiation leukaemogenesis can now be checked by direct experiment since it has proved possible to induce acute myeloid leukaemia (AML) systematically in an experimental animal by a variety of ionizing radiations, X-rays (Major & Mole 1978, Mole & Meldrum 1981), $\gamma$-rays (Mole & Major 1983) and fission neutrons (Mole & Davids 1982). The induction process for X-rays is found to increase not linearly but according to the square of the radiation dose.

The form of the dose-response for ionizing radiation is not simply of interest for radiological protection. The geometry of the ionizing tracks in tissue may allow inferences to be made about the physical size of the target for radiation action. The X-ray dose response for AML in CBA/H mice suggests that the target for leukaemia induction is larger than a single cell.

Furthermore the dose-response for observed frequency of malignant disease after exposure to ionizing radiation cannot be taken to represent the dose-response for induction. What is observed *in vivo* is the net result of two opposing processes at the cellular level, each of which is a dose-dependent consequence of the same radiation exposure, induction on the one hand and cellular inactivation on the other hand. Cellular inactivation prevents the development of overt malignancies which would otherwise follow induction. The quantitative consequences of this interaction were first examined by Gray (1965) and later by Mole (1975, 1979*a*, 1983). In principle, corresponding interactions need to be taken into account when assessing the quantitative aspects of induction of malignant disease by other agents. These quantitative aspects are now becoming as important for chemical agents as they have been for several decades for ionizing radiation.

### Materials and methods

CBA/H mice have been maintained in this laboratory by brother-sister mating for over 30 years. Brief exposures to 250 kVp X-rays (Corp, 1957) were given to CBA/H males in the age range 100±8 days. In most experiments full-sib brothers were caged together from birth until death (one cage per litter) except when separated in order to be irradiated. Radiation or other treatments were

Correspondence: R.H. Mole, Heath Barrows, Bayworth Lane, Boars Hill, Oxford OX1 5DF.

*Visitor.

Received 14 July 1982; accepted 25 October 1982.

0007-0920/83/020285-07 $02.00

© The Macmillan Press Ltd., 1983

allocated by a randomising procedure usually with the restriction that no 2 mice from the same litter should receive the same treatments. In a few experiments mice were randomised between cages (4–5 mice per cage) and the cage treatment was allocated at random. The present report deals with animals which received single X-ray exposures at 0.50–0.55 Gy $min^{-1}$ or at 5.5 Gy $min^{-1}$.

Five–12 mice from different litters received a given dose on the same day. Replicates at intervals of one to a few weeks and commonly spread over several years during the period September 1972–February 1980 allowed the numbers of animals receiving a given dose to build up to an average of nearly 100 and tended to smooth out possible temporal variations in response associated with uncontrolled factors such as the detailed composition of the food. No such variability was in fact recognised.

No epidemics or other untoward events affected survival. No experiment or batch of animals has been excluded on the grounds that results were unsatisfactory.

*Diagnosis of myeloid leukaemia*

Each cage was examined daily for sick or dead mice. Each animal received a comprehensive autopsy with special reference to neoplasms obvious or suspected. Sternum and liver were routinely taken for histological examination and when free from obvious infiltration were regarded as proof that the mouse did not have leukaemia.

The macroscopic appearance of AML in the mouse is usually characteristic or highly suggestive (Major & Mole 1978). The spleen is enlarged uniformly 5–20 fold. Its colour varies with the degree of anaemia from slightly blue or purple with a mottled surface to pale pink. Lymph nodes may be slightly enlarged. The liver is enlarged up to 3–4 fold and commonly obviously infiltrated. Additional tissues were taken from every such animal. Diagnosis was based on a minimum of gross infiltration of liver, extramedullary infiltration of muscle around bone and almost complete replacement of normal marrow, all with clearly abnormal and myeloid cells. A variable proportion of the marrow could be pyknotic or acellular. Cases of myeloid leukaemia with positive histology in liver and bone but wholly unsuspected at autopsy have been very uncommon. The major diagnostic difficulty has been post-mortem degeneration which rarely prevents recognition of gross infiltration but may entail prolonged search to identify the cellular type. The characteristic ring nucleus of the rodent myelocyte is then of major help in making a positive diagnosis of myeloid leukaemia in circumstances where distinctions between non-myeloid cell types would be impossible to make. Cases not positively identifiable as myeloid have been excluded from consideration of dose responses.

A substantial proportion of mice with myeloid leukaemia but by no means all show a clinically evident anaemia and a progressive loss of weight. Blood examination at this stage allows a diagnosis to be made in advance of autopsy. Periodic routine blood counts on every animal have not been done in any experiment so far.

*Calculation of dose response*

No upper limit is postulated for the number of different leukaemias which might be induced in any given individual: this cannot be determined experimentally because of the movement around the body and the mixing of leukaemia cells from an early stage of leukaemia development. Each calculated dose-response is derived by the method of maximum likelihood, is based on leukaemia frequency stated in terms of $p$ the fraction of animals at risk which developed myeloid leukaemia, and is quantal in character i.e. is calculated from the proportion of animals without leukaemia because $(1-p)=e^{-y}$ where $y$ is the mean number of leukaemias per animal and control frequency is zero. The induction coefficients $a$ are risks for a dose of 0.01 Gy to an individual animal whereas $\lambda$ is the fractional probability of cell inactivation for a dose of 0.01 Gy. If the corresponding probability of transformation per cell for a dose of 0.01 Gy is $\mu$, $a=\mu N$ where $N$ is the (unknown) number in the individual of those primitive haematopoietic cells potentially transformable into "mother cells" of myeloid leukaemia. Hence $\mu$ is orders of magnitude smaller than $\lambda$ (Mole, 1975). No corrections have been made for differences according to radiation dose in overall survival of non-leukaemic individuals, differences which, if they existed, might be expected to bias leukaemia frequencies in a dose-dependent manner.

### Results

The experiments originally reported (Major & Mole, 1978) have continued, the number of mice at a given dose being increased and the range of doses enlarged with the aim of providing degrees of freedom sufficient for statistical testing of theoretical models. Myeloid leukaemia was found after each of 9 different X-ray doses in the range 0.25–4.5 Gy inclusive (Table I). No case was found after the largest dose 6.0 Gy (not large enough to kill acutely) or in more than 800 unirradiated controls accumulated during the overall experimental period.

**Table I** X-ray dose and frequency of myeloid leukaemia

| Calendar years exposure (overall) | 1972–80 | 1977–79 | 1977–79 | 1974–80 | 1977–79 | 1972–80 | 1977–78 | 1975–79 | 1972–78 | 1972–80 | 1974 |
|---|---|---|---|---|---|---|---|---|---|---|---|
| Dose (Gy)* | 0 | 0.25 | 0.50 | 0.75 | 1.00 | 1.50 | 2.00 | 2.50 | 3.00 | 4.50 | 6.00 |
| No. of mice† | 800+ | 130 | 133 | 100 | 53 | 78 | 40 | 88 | 118 | 169 | 42 |
| Myeloid leukaemia | | | | | | | | | | | |
| No. cases | 0 | 1 | 7 | 5 | 5 | 11 | 4 | 13 | 25 | 20 | 0 |
| % | 0 | 1 | 5 | 5 | 9 | 14 | 10 | 15 | 21 | 12 | 0 |
| Median survival of mice without myeloid leukaemia (lunar months) | | | | | | | | | | | |
| Irradiated | — | 24 | 24 | 23 | 24 | 23 | 21 | 24 | 23 | 22 | 20 |
| Contemporary controls‡ | 24 | 23 | 24 | 25 | 23 | 25 | 23 | § | 24 | 24 | 22 |

*Dose rate 0.50–0.55 Gy $min^{-1}$.

†Excluding a few animals dying within 100 days of the starting date of an experiment.

‡The values are not for independent batches of controls since unirradiated mice were usually controls for more than one X-ray dose.

§Half the mice were irradiated in the winter of 1975–76 and their contemporary controls showed a uniquely long survival, median 30 lunar months. No other X-ray exposures were made at that time. The other mice in the dose group were exposed in 1977 and 1979 when the median survival of their contemporary controls was 24 months.

The distribution of cases with time after irradiation is shown in Figure 1. The earliest cases occurred at 8–10 months after irradiation and thereafter the number of new cases of myeloid leukaemia in a given period of time remained roughly constant until the number of surviving mice began to be substantially reduced by natural causes. A very similar picture was found with fission neutrons (Mole & Davids 1982). There was little, if any, correlation between magnitude of X-ray dose and distribution of cases with time since exposure (Figure 1).

The survival of irradiated mice which did not develop myeloid leukaemia was closely similar to that of the unirradiated except at the very highest doses. The median survival time for each separate

**Figure 1** Temporal distribution of cases of AML in male CBA/H mice after a single brief X-ray exposure according to dose. 0.25–1.50 Gy is in the region of the dose-response where leukaemia frequency increases with dose, 2.0–3.0 Gy lies in the plateau region and 4.50 Gy is in the region where leukaemia frequency decreases with dose (Figure 2).

dose group was close to that for its contemporary controls (Table 1). The cumulative mortality curve after 6.0 Gy and in unirradiated controls is illustrated in Major & Mole (1978).

*Dose response*

*Competition between induction and loss of cells by inactivation* The observed dose-response for any form of malignant disease induced by ionizing radiation must depend on the interaction of 2 processes, cell transformation and inactivation (Gray 1965; Mole, 1975, 1979*a*, 1983). Each of these can be caused by ionizing radiation and both are relevant because, unless a transformed cell escapes inactivation, it cannot continue to divide indefinitely. Without retention of this clonogenic ability no overt malignancy can develop from a transformed cell or focus of cells. Therefore the dose-dependent frequency of myeloid leukaemia observed *in vivo* after exposure to ionizing radiation will not represent the dose-response for induction but rather the net consequence of two independently dose-dependent processes, that for cell transformation i.e. induction, and that for inactivation of potentially transformable cells and of cells that have been transformed. Inactivation of both kinds of cells needs to be considered for any radiation exposure spread out over a finite period of time.

*Radiobiological considerations* The currently accepted general hypothesis is that cellular effects of ionizing radiation are quantitatively dependent on a polynomial in dose $D$, $a_1D+a_2D^2+\ldots$, as illustrated in a mass of observations on inactivation of cells and on mutation, both genetic and chromosomal. This applicability was taken for granted in the latest U.S.A. review of radiation carcinogenesis in man, the BEIR Report of 1980.

The probability of cell transformation per unit absorbed dose of ionizing radiation must be very much smaller than the probability of cell inactivation (Mole, 1975). This leads directly to the hypothesis (Mole 1975, 1983) that the observed frequency of induced malignant disease $y$ will depend on dose according to

$$y=(a_1D+a_2D^2+\ldots)\,e^{-(\lambda_1D+\lambda_2D^2+\ldots)}$$

where the coefficients $a$ refer to induction and the coefficients $\lambda$ refer to cellular inactivation. A possible justification for the polynomial is the idea that some single ionization tracks are capable of effective action on their own and that traversal of a cell by 2 or more tracks provides additional means for damage to cells or targets within cells.

*The observed dose-response for X-ray induced acute myeloid leukaemia in male CBA/H mice: X-ray exposures at constant dose rates* Table II gives the results of fitting the above equation and simplified versions of it to the observations listed in Table I. All 5 equations yield statistically acceptable fits but only (i) gives physically meaningful values to all its parameters. This equation (i) postulates cell transformation, i.e. induction of leukaemia, according to $D^2$ together with a simple exponential

**Table II** Numerical values of parameters mean ±s.e. for dose-responses for induction of myeloid leukaemia in male CBA/H mice by single brief exposures to 250 kVp X-rays giving tissue doses in the range 0.25–6.0 Gy

| *Dose response* | $a_1$ | $a_2$ | $\lambda_1$ | $\lambda_2$ | *P for goodness of fit* |
|---|---|---|---|---|---|
| (i) $a_2D^2e^{-\lambda_1D}$ | | $2.4\pm0.5$ $10^{-5}$ | $8.25\pm0.74$ $10^{-3}$ | | 0.44 |
| (ii) $(a_1D+a_2D^2)e^{-\lambda_1D}$ | $-1.4\pm5.4$ $10^{-4}$ Neg. n.s. | $2.5\pm1.0$ $10^{-5}$ | $8.4\pm1.0$ $10^{-3}$ | | 0.34 |
| (iii) $a_2D^2e^{-(\lambda_1D+\lambda_2D^2)}$ | | $1.6\pm0.6$ $10^{-5}$ | $5.0\pm3.0$ $10^{-3}$ n.s. | $5.9\pm5.2$ $10^{-6}$ n.s. | 0.38 |
| (iv) $(a_1D+a_2D^2)$ $e^{-(\lambda_1D+\lambda_2D^2)}$ | $5.6\pm2.1$ $10^{-4}$ | $4.7.10^{-11}$ $\pm2.3.10^{-4}$ n.s. | $-5.7.10^{-3}$ $\pm4.1.10^{-1}$ Neg. n.s. | $1.7\pm0.5$ $10^{-5}$ | 0.42 |
| (v) $a_1De^{-\lambda_2D^2}$ | $1.06\pm0.16$ $10^{-3}$ | | | $6.9\pm1.3$ $10^{-6}$ | 0.38 |

$D$ = tissue dose, Neg. indicates that the mean value for the parameter is not positive.
n.s. = statistically not significantly different from zero.

cell survival response (i.e. without shoulder) for the primitive haematopoietic cells presumed to be those susceptible to transformation into the ancestral "mother" cells from which all the leukaemia cells in an affected individual develop. Figure 2 illustrates the fit of equation (i) to the data points.

**Figure 2** Myeloid leukaemia frequency % with ±80% binomial confidence limits after whole body exposure of male CBA/H mice to 250kVp X-rays giving tissue doses in the range 0.25–6.0 Gy. No case has been seen in over 800 unirradiated controls so far fully examined. The fitted curve is $aD^2 e^{-\lambda D}$ (Table II).

According to current radiobiological concepts an induction process linear in dose should form a substantial part, if not the whole, of the leukaemogenic effect of 0.25–0.50 Gy of X-rays received in a brief exposure of 0.5–1 min. However the linear coefficient $a_1$ (equation (ii) Table 2)) is negative and therefore physically unrealistic. Moreover including a linear term for induction reduced the precision of the values for the other parameters (Table II (i) and (ii)), contrary to what might be expected if there was truly such a linear component in the induction process.

Equation (v) does not have a straightforward radiobiological justification since no known cell survival response depends solely on $D^2$ and is therefore rejected. The choice between equations (i) and (v) may become important if the attempt is made to infer risks after low doses. For equal frequencies of leukaemia after 0.75 Gy (as given by the numerical values in Table II) the inferred frequency after 0.01 Gy according to equation (v) is 45 × larger than according to equation (i).

The fit of equation $y = aDe^{-\lambda D}$ which was satisfactory for fission neutrons (Mole & Davids 1983) is poor for X-rays ($P = 0.066$).

Equation (i) is therefore regarded as the only statistically, physically and radiobiologically satisfactory fit to the observations.

*X-ray dose response for brief exposure 8–65s long* A strict application of the radiobiological principles exemplified in the polynomial utilised to provide a dose-response requires that the duration of exposure is held constant while radiation dose varies. In the experiments, however, dose rate was held constant and the duration of irradiation over the dose range 0.25–6.0 Gy varied 24-fold from 27 sec to 11 min. The degree of error thus introduced depends on the rate of repair of the radiation damage in relation to the duration of exposure (see Lea, 1955 in the context of chromosome rejoining time and the yield of translocations).

Observations on myeloid leukaemia induction by X-rays at 5.5 Gy min$^{-1}$ have still to be completed. The replicates completed so far with 6 doses in the range 0.75–6.0 Gy together with the data on 0.25 and 0.5 Gy at 0.5–0.55 Gy min$^{-1}$ (Table I) provide a dose-response for exposures of duration 8–65 sec, where the duration of exposure is less systematically correlated with magnitude of dose, and the overall variation in duration of exposure is smaller, than for the data of Table I, and where the time available for repair processes to operate during an exposure is less or much less than for the doses 0.75–6.0 Gy at 0.50–0.55 Gy min$^{-1}$. Equation (i) is fitted just as satisfactorily ($P = 0.40$) and $aDe^{-\lambda D}$ is clearly rejected ($P < 0.01$) as, according to the criteria used above, are equations (ii)–(v) also. These additional observations serve to confirm the conclusion of the previous section that the only equation among those tested which provides a satisfactory description of the experimental data is equation (i). As for the data of Table I the linear co-efficient for induction in equation (ii) is negative.

*Additional considerations* In many experiments on radiation carcinogenesis there are serious problems of interpretation when the frequency of neoplasms other than that under investigation is also affected in a dose-dependent manner, especially if the timing of their occurrence is earlier than or coincident with the neoplasm under investigation. Thus in work on induction of chronic myeloid leukaemia in male X-rayed RF/Un mice (Upton *et al.*, 1970) there was a progressive reduction with increase in dose in "other leukaemias" from the control value of 32% to 15% after 4.5 Gy and an increase in frequency of thymic lymphoma from 4–16% (with latent periods shorter than for chronic myeloid leukaemia). Control male CBA/H mice have 2–3% non-myeloid "leukaemia" (mostly occurring late in life) and this is increased after 4.5 and 6.0 Gy X-rays. Eventually small corrections will need to be made for such competing causes of death and for the small differences in the distribution of survival time between groups of irradiated mice without myeloid leukaemia. Consideration will also have to be given to the omission of the earlier observations after 2.5 Gy when control survival was so unusually long (Table 1 footnote §). Such corrections should not affect the broad conclusions drawn from the curvilinearity of the observations as reported here.

### Discussion

The dose-response for the observed frequency of myeloid leukaemia in male CBA/H mice after whole-body X-irradiation with doses in the range 0.25–6.0 Gy is clearly highly curvilinear (Figure 2) and this of itself signifies that no simple explanation is possible. Only one simple polynomial expression $aD^2 e^{-\lambda D}$ was found to provide both a statistically satisfactory fit to the observations and values for its parameters which were positive and significantly greater than zero and radiobiologically reasonable. Such a dose response is also capable of interpretation in terms of straightforward biological and cellular considerations. Induction, i.e. transformation of primitive haematopoietic cells into "mother cells" of leukaemia clones, is represented by $aD^2$ and survival of potentially transformable and of transformed cells by $e^{-\lambda D}$.

This survival function would be expected on general grounds to be closely similar to that of irradiated haematopoietic stem cells when measured by spleen colony assays or by the ability to rescue animals from otherwise certain death following supralethal exposure of the whole animal. The values of $\lambda$ found in such experiments are closely similar to the value of $\lambda$ inferred solely from the observations on myeloid leukaemia. Mean values for 17 measurements of $\lambda$ for femoral marrow CFU-S in a variety of mice irradiated by 200–300 kVp X-rays *in vivo* were in the range 0.0095–0.0161 cGy$^{-1}$ (Hendry & Lord 1983). These may be compared with the value of 0.007–0.01 cGy$^{-1}$ inferred exclusively from observations on myeloid leukaemia.

An induction process proportional to $D^2$ implies that two targets, each affected by irradiation, must interact. The classical examples are the exchanges of chromosomal material which follow damage to two different chromosomes and are observed as reciprocal translocations, or which follow damage to two DNA strands within one and the same chromosome and are observed later on in the cell cycle as chromosomal "intra-changes". When the ionizing tracks in a given tissue volume are numerous enough ($D$ is large) the targets are damaged independently by two different ionizing tracks and the dose response is proportional to $D^2$. When the dose is low enough the number of ionizing tracks in a given small volume becomes so low that any effect which results is the consequence of a single ionizing track traversing both the targets in question and the dose response is then proportional to $D$. Thus in general the dose response over a wide range of dose $= a_1D + a_2D^2$ as is also found experimentally for inactivation of mammalian cells. Dose $D$ for a given quality of radiation is directly proportional to the average number of ionizing tracks in a tissue volume.

The ratio $a_1/a_2$ gives the value of dose $D$ at which the effect of the linear component $a_1D$ equals the effect of the dose-squared component, $a_2D^2$. For mutation or inactivation of mouse and Chinese hamster cells the observed values of $a_1/a_2$ lie in the range 0.7–14 Gy, for inactivation of cultured human cells in the range 2.0–25 Gy and for chromosome aberration induction in cultured human blood lymphocytes in the range 0.31–1.9 Gy with a median value of about 1 Gy (Brown 1977, Fertil *et al.*, 1980).

For induction of myeloid leukaemia *in vivo* $a_1/a_2$ has a physically unrealistic negative value (Table 2 equation (ii)). The SE of this mean value is large and its upper 95% confidence limit is 1.29 Gy, within the range of values for chromosome aberrations in human blood lymphocytes but at or below the lower limit for the other phenomena listed. However 1.29 Gy is an extreme possibility. The corresponding upper 95% confidence limit for the part-completed observations after brief exposures 8–65 sec. in duration is 0.08 Gy. It is thus a reasonable conclusion that the size of the target for induction of myeloid leukaemia is almost certainly very different from, i.e. much larger than, that for chromosome aberrations or cell inactivation.

The hypothesis that myeloid leukaemia induction by ionizing radiation and perhaps radiation carcinogenesis in general is the consequence of damage to 2 adjoining cells is discussed more fully elsewhere and is shown to provide a potentially useful framework of understanding (Mole, 1983). Endocytotic hypotheses for carcinogenesis imply that incorporation of "foreign" genetic material into the genome is essential (Mole, 1979*a*). It is tentatively envisaged that substantial fragments of nuclear DNA liberated by dissolution of one cell damaged by irradiation are transferred into an immediately neighbouring cell damaged by irradiation in a manner which facilitates pinocytosis of these fragments and then their incorporation into its genome. It is clear that evidence for such an hypothesis involves a great deal more than analysis of a dose-reponse for carcinogenesis *in vivo*.

For exposures protracted over several weeks the dose response for leukaemia is very similar after part-body X-ray therapy of human subjects with ankylosing spondylitis (Smith & Doll, 1982) and after whole-body $\gamma$-ray irradiation of male CBA/H mice (Mole & Major 1983) but is quite unlike that shown in Figure 2. In both these cases the dose response for protracted exposure is very flat and comparatively little dependent on dose. There must be factors important in leukaemogenesis by ionizing radiation waiting to be identified and identifiable perhaps only by experimental analysis *in vivo*. When these factors are understood it will be easier to assess the validity of extrapolations from equations fitted to observations on relatively heavily-irradiated individuals, whether mice or men, to the circumstances of long protracted low level exposure. Moreover it may well be that different categories of radiation-induced cancer do not necessarily have the same form of dose-response (Mole 1979*b*, 1983, Mole & Davids 1982).

The Medical Research Council provided financial support to R.H.M. to pursue this project and for this we are grateful.

**References**

BEIR, COMMITTEE ON BIOLOGICAL EFFECTS OF IONIZING RADIATIONS (1980). *The effects on populations of exposure to low levels of ionizing radiation: 1980*. Washington D.C. National Research Council, National Academy Press.

BROWN, J.M. (1977). The shape of the dose-response curve for radiation carcinogenesis. *Rad. Res.*, **71**, 34.

CORP, M.J. (1957). Whole body X-irradiation of experimental animals. *Phys. Med. Biol.*, **1**, 370.

FERTIL, B., DESCHAVANNE, P.J., LACHET, B. & MALAISE, E.P. (1980). *In vitro* sensitivity of six human cell lines. *Radiat. Res.*, **82**, 297.

GRAY, L.H. (1965). Radiation biology and cancer. In *Cellular Radiation Biology*. Baltimore, Williams and Wilkins. p. 7.

HENDRY, J.H. & LORD, B.I. (1983). The analysis of the early and late response to cytotoxic insults in the haematopoietic cell hierarchy. In *Cytotoxic Insult to Tissue*, (Eds. Potten & Hendry). Edinburgh, Churchill Livingstone (in press).

LEA, D.E. (1955). *Actions of Radiations on Living Cells 2nd Edn*. Cambridge University Press: p. 225.

MAJOR, I.R. & MOLE, R.H. (1978). Myeloid leukaemia in X-ray irradiated CBA mice. *Nature*, **272**, 455.

MOLE, R.H. (1975). Ionizing radiation as a carcinogen: practical questions and academic pursuits. *Br. J. Radiol.*, **48**, 157.

MOLE, R.H. (1979*a*). Carcinogenesis by Thorotrast and other sources of irradiation especially α-emitters. *Environ. Res.*, **18**, 192.

MOLE, R.H. (1979*b*). RBE for carcinogenesis by fission neutrons. *Health Phys.*, **36**, 463 and **37**, 605.

MOLE, R.H. (1983). Dose response relationships. In *Radiation Carcinogenesis: Epidemiology and biologic significance*, (Eds. Boice & Fraumeni). New York: Raven Press in press.

MOLE, R.H. & DAVIDS, J.A.G. (1982). Induction of myeloid leukaemia and other tumours in mice by irradiation with fission neutrons. In *Neutron Carcinogenesis* (Eds. Broerse & Gerber). Luxemburg: CEC Directorate, General Information, Marketing and Innovation, p. 31.

MOLE, R.H. & MAJOR, I.R. (1983). Myeloid leukaemia frequency after protracted exposure to ionizing radiation: Experimental confirmation of the flat dose-response found in ankylosing spondylitis after a single treatment course with X-rays. *Leuk. Res.*, **7**, (in press).

MOLE, R.H. & MELDRUM, R.A. (1981). Myeloid leukaemia induction in mice by brief exposure to X-rays and fission neutrons. *Leuk. Res.*, **5**, 173.

SMITH, P.G. & DOLL, R. (1982). Mortality among patients with ankylosing spondylitis after a single treatment with X-rays. *Br. Med. J.* **i**, 449.

UNITED NATIONS SCIENTIFIC COMMITTEE ON THE EFFECTS OF ATOMIC RADIATION (1977). Radiation carcinogenesis in man. Appendix G, *Sources and Effects of Ionizing Radiation*, New York: United Nations.

UPTON, A.C., RANDOLPH, M.L. & CONKLIN, J.W. (1970). Late effects of fast neutrons and gamma-rays in mice as influenced by the dose rate of irradiation: Induction of neoplasia. *Radiat. Res.*, **41**, 467.